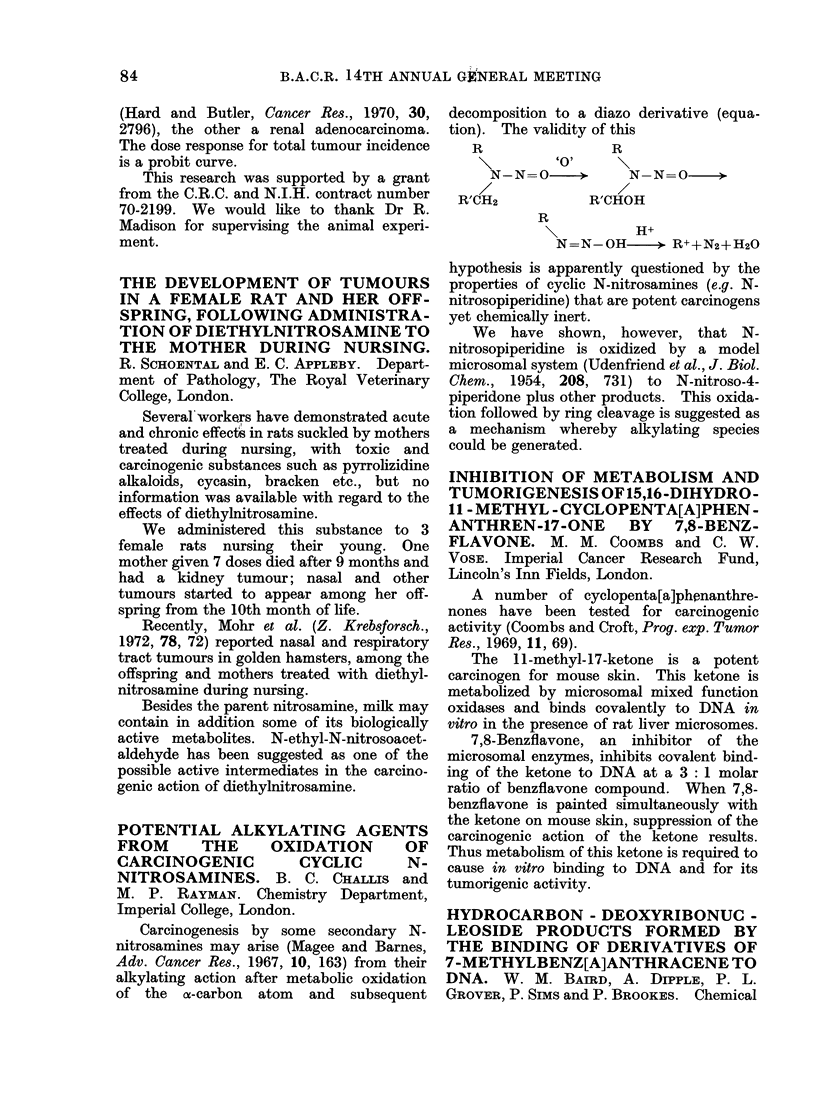# The development of tumours in a female rat and her offspring, following administration of diethylnitrosamine to the mother during nursing.

**DOI:** 10.1038/bjc.1973.100

**Published:** 1973-07

**Authors:** R. Schoental, E. C. Appleby


### THE DEVELOPMENT OF TUMOURS IN A FEMALE RAT AND HER OFFSPRING, FOLLOWING ADMINISTRATION OF DIETHYLNITROSAMINE TO THE MOTHER DURING NURSING.

R. SCHOENTAL and E. C. APPLEBY. Department of Pathology, The Royal Veterinary College, London.

Several workers have demonstrated acute and chronic effects in rats suckled by mothers treated during nursing, with toxic and carcinogenic substances such as pyrrolizidine alkaloids, cycasin, bracken etc., but no information was available with regard to the effects of diethylnitrosamine.

We administered this substance to 3 female rats nursing their young. One mother given 7 doses died after 9 months and had a kidney tumour; nasal and other tumours started to appear among her offspring from the 10th month of life.

Recently, Mohr *et al.* (*Z. Krebsforsch.*, 1972, **78**, 72) reported nasal and respiratory tract tumours in golden hamsters, among the offspring and mothers treated with diethylnitrosamine during nursing.

Besides the parent nitrosamine, milk may contain in addition some of its biologically active metabolites. N-ethyl-N-nitrosoacetaldehyde has been suggested as one of the possible active intermediates in the carcinogenic action of diethylnitrosamine.